# Asthma-related deaths

**DOI:** 10.1186/s40248-016-0073-0

**Published:** 2016-10-12

**Authors:** Gennaro D’Amato, Carolina Vitale, Antonio Molino, Anna Stanziola, Alessandro Sanduzzi, Alessandro Vatrella, Mauro Mormile, Maurizia Lanza, Giovanna Calabrese, Leonardo Antonicelli, Maria D’Amato

**Affiliations:** 1 Division of Respiratory and Allergic Diseases, Department of Chest Diseases, High Speciality “A. Cardarelli” Hospital, Napoli, Italy; 2 Department of Medicine and Surgery, University of Salerno, Salerno, Italy; 3 First Division of Pneumology, High Speciality Hospital ‘V. Monaldi’ and University ‘Federico II’ Medical School Naples, Napoli, Italy; 4 Second Division of Pneumology, High Speciality Hospital ‘V. Monaldi’ and University ‘Federico II’ Medical School Naples, Napoli, Italy; 5 Service of Immunoallergology, University Hospital “Ospedali Riuniti”, Ancona, Italy

**Keywords:** Asthma-deaths, Asthma mortality trends, Inhaled corticosteroids, Near fatal asthma

## Abstract

Despite major advances in the treatment of asthma and the development of several asthma guidelines, people still die of asthma currently. According to WHO estimates, approximately 250,000 people die prematurely each year from asthma. Trends of asthma mortality rates vary very widely across countries, age and ethnic groups. Several risk factors have been associated with asthma mortality, including a history of near-fatal asthma requiring intubation and mechanical ventilation, hospitalization or emergency care visit for asthma in the past year, currently using or having recently stopped using oral corticosteroids (a marker of event severity), not currently using inhaled corticosteroids, a history of psychiatric disease or psychosocial problems, poor adherence with asthma medications and/or poor adherence with (or lack of) a written asthma action plan, food allergy in a patient with asthma. Preventable factors have been identified in the majority of asthma deaths. Inadequate education of patients on recognising risk and the appropriate action needed when asthma control is poor, deficiencies in the accuracy and timing of asthma diagnosis, inadequate classification of severity and treatment, seem to play a part in the majority of asthma deaths. Improvements in management, epitomized by the use of guided self-management systems of care may be the key goals in reducing asthma mortality worldwide

## Background

Asthma is a serious global health problem affecting at least 300 million people, with a high global burden of disability [1–4]. Despite major advances in the treatment of asthma and the development of several asthma guidelines over the past decades, people still die of asthma currently [1]. According to WHO estimates, approximately 250,000 people die prematurely each year from asthma [3].

## Main body

### Trends of asthma mortality

Recorded asthma mortality rates vary very widely across countries (http://dati.istat.it/) [5–23]. The estimates of asthma deaths are affected by several factors. One of these factors is precisely the correct estimation of mortality from asthma. As the national mortality statistics are derived from death certificates, inaccurate coding due to lack of knowledge of the clinical history may lead to imprecise estimation of mortality rates from asthma [24]. Then it should be considered that international mortality statistics for asthma are limited to those countries reporting a full set of causes of death.

According to health statistics, asthma mortality has significantly decreased worldwide over past decades. The American Lung Association reported information, available from national and state-based surveys on the mortality due to asthma 1999–2009 [8, 9]. After a long period of steady increase, evidence suggests that asthma mortality and health care utilization rates continue to plateau and/or decrease. The number of deaths due to asthma in 2009 was approximately 27 % lower than the number of deaths seen in 1999 [9]. The number and rate of hospital discharges have both decreased 24 % between 2003 and 2010 [10]. In Japan over 7000 patients died every year in the 1970s, while 1874 patients died of an asthma attack in 2012 [11, 12]. Asthma-related mortality in Europe has significantly decreased, from 6287 deaths in 1985 to 1164 in 2012 (−80 %) [5].

In Italy deaths from asthma have declined in recent decades, passing from 1500 in 1995 to 429 in 2013 [6] (http://dati.istat.it/).

Among children asthma mortality rates have been declining since 2000 in countries where they have been measured (Table 1).

**Table 1:** Trends in asthma mortality

In recent decades mortality from asthma has declined worldwide, nevertheless the number of asthmatic patient who die still remains very high.	

The introduction of inhaled corticosteroids (ICS) in the treatment of asthma, in the early 1970s, played a key role in reducing asthma mortality. It’s widely recognized that the widespread and progressive increase in the use of ICS therapy over the past 20 to 30 years has decreased asthma mortality and morbidity [25], further, ICS have been found to reduce asthma hospitalization rates by as much as 80 % [26].

In contrast, several evidences show that inappropriate use of beta agonist drugs may increase the risk of asthma mortality. Short-acting-agonists (SABA) can induce tolerance and increase airway hyperresponsiveness [27, 28]. Excess use of SABA has also been found to increase the risk of asthma death [29]. In the past, during the mid-to-late 1960s, the introduction of high dose isoprenaline inhalers as an asthma reliever medication, which can have toxic effects on the heart during acute asthma attacks, was associated with an approximately 50 % increase in asthma death rates among 5–34 year olds [14]. When these medications were withdrawn, the 1960s epidemic of asthma deaths subsided. Successively, during the mid-1980s, an increase of approximately 38 % in asthma death was associated with the widespread use of fenoterol, another inhaled asthma medication with potential cardiac toxicity [14]. Recently, a metanalysis has been conducted of asthma deaths in randomised controlled clinical trials that compared salmeterol with a non-LABA comparator treatment in asthma. Salmeterol monotherapy in asthma increases the risk of asthma mortality and this risk is reduced with concomitant ICS therapy [29].

### Risk factors for asthma-related deaths

Several risk factors have been associated with asthma mortality, including a history of near-fatal asthma requiring intubation and mechanical ventilation, hospitalization or emergency care visit for asthma in the past year, currently using or having recently stopped using oral corticosteroids (a marker of event severity), not currently using inhaled corticosteroids, a history of psychiatric disease or psychosocial problems, poor adherence with asthma medications and/or poor adherence with (or lack of) a written asthma action plan, food allergy in a patient with asthma [1, 30–36].

Near-fatal asthma (NFA) and fatal asthma represent the most severe clinical presentations of asthma. Near fatal asthma (NFA) is described as acute asthma associated with a respiratory arrest or arterial carbon dioxide tension greater than 50 mmHg, with or without altered consciousness requiring mechanical ventilation [4, 37–39]. In the NFA the most important pathophysiological events that lead to death are cardiac arrhythmias and asphyxia followed by complications of invasive mechanical ventilation such as barotrauma and ventilator-associated pneumonia. In a study of asthma patients admitted with a near-fatal episode, two-thirds of subsequent severe attacks or deaths occurred within 1 year of the previous life-threatening admission [39].

A trigger of fatal and near fatal asthma was identified in mould sensitivity [40–45]. Fungi are very common in the environment and airborne spores are a normal component of the outdoor air, so the respiratory exposure is almost constant. Many different fungi have been implicated in exacerbations of asthma, *Alternaria spp., Cladosporium spp.* and *Aspergillus fumigatus* seem to be the most important [40–47].

It was observed that the prevalence of sensitisation to moulds (*Alternaria alternate* or *Cladosporium herbarum*, or both) increased with increasing severity of asthma [40].

Sensitisation to moulds has been associated with increased asthma severity and death, hospital admission and intensive care admissions in adults and with increased bronchial reactivity in children [40–48]. In a retrospective study of 11 asthmatic patients who had a respiratory arrest, 10 out of 11 were skin-test-positive for *Alternaria alternate* [42].

Black et al. reported that twenty out of 37 patients (54 %) admitted to the ICU for asthma had a positive skin test for one or more fungal allergens [43]. Targonsky et al. reported that, during the pollen season, mean concentrations of mould spores, but not of tree, grass, or ragweed pollen, were significantly higher on the days when there were deaths related to asthma than on the days when no such deaths occurred [44].

It remains to be clarified the pathogenetic mechanisms by which certain mould allergens produce more severe airways disease than other common allergens. A possible explanation is that fungi, compared to other allergens, have the additional ability to actively germinate and infect the host skin or attempt to colonise the respiratory tract [41]. It was suggested that mould allergens acting in concert with other nonallergen proteins or toxins produce an enhanced host response [41].

Several factors were identified as contributing factors in asthma deaths [7–19, 49–65].

With the regard to age, Bellia et al. followed up 1233 ambulatory patients aged > 65 years with a diagnosis of asthma or chronic non respiratory conditions and showed that asthma in the elderly was associated with higher mortality rate, although this condition was not an independent risk factor [16]. Differently, age was a significant predictor of in-hospital mortality in asthma patients, however a higher burden of underlying comorbidities in elderly patients could likely contribute to higher in-hospital mortality [13, 18]. Asthma hospitalization during the winter months was also significantly predictive of increased in-hospital mortality, probably because winter months were associated with a concurrently higher prevalence of respiratory viral infections, including influenza [13]. Elderly patients have a higher incidence of asthma exacerbations and asthma-related deaths during the midwinter months [13, 19].

Among children, the International Study of Asthma and Allergies in Childhood (ISAAC) found a significantly positive correlation between childhood asthma mortality and the prevalence of more severe asthma symptoms in both 6–7 year olds (29 countries) and 13–14 year olds (38 countries) [48].

In a limited number of cases, exercise was described as a trigger of fatal asthma attacks specially in children and adolescents. In a preliminary investigation of asthma mortality in US school between 1990 and 2003, 38 asthma deaths were reported [49]. Of the fatal asthma attacks, 16 (42 %) occurred while the children were participating in a physically active event [49].

In a report of asthma deaths during sports from 1993 until 2000, 61 cases were analyzed [50].

White males 10 to 20 years old were most at risk of dying from asthma during sports. Sudden fatal asthma exacerbations occured in both competitive and recreational athletes. Athletes who played for either a professional or school team accounted for 57 % of the total confirmed deaths. The rest (43 %) were people who competed recreationally. 51 % of the victims died in the middle of playing their sport [50].

In a 25-year follow-up study in a large cohort of asthmatic patients, age, level of lung function, degree of bronchodilator reversibility, peripheral blood eosinophil count, and previous acute hospital contacts for asthma at enrollment were identified as long-term risk factors for subsequent death from asthma. After adjusting for age and level of FEV_1_ % predicted, not allergic asthma was associated with a higher risk of death from asthma (RR, 1.9; 95 % CI, 1.1–3.2; *p* 5 .001) [15]. No significant association was found between smoking habits or self-reported symptom severity, and subsequent death from asthma [15]. Lifetime tobacco consumption increases the risk of death from asthma in a previous study [7]. Almind et al. previously showed an excess mortality among male asthmatic smokers [51], whereas a Finnish study found no association between mortality and smoking among people with asthma [52].

Regarding to ethnic differences in asthma, there are only a limited number of within-country investigations. Data from the US have shown that African-Americans are at increased risk of exacerbations, hospital attendances, near death episodes, and mortality [17, 53–55]. This has led to recent substantial investments ($23 m) to reduce asthma disparities through the Patient-Centered Outcomes Research Institute [56]. A previous systematic review and meta-analysis investigating ethnic variations in asthma outcomes in the UK found that South Asians (odds ratio (OR) = 2.9; 95 % CI, 2.4–3.4) and Blacks (OR = 2.1; 95 % CI, 1.8–2.5) were at substantially increased risk of hospital admission from asthma when compared to White European-origin populations [58].

Recently, a prospective study has examined the relationship between reproductive factors (parity and age at first birth) and the risk of asthma death. There is a significant protective effect of parity on the subsequent risk of death from asthma because risk of death from asthma increased with increasing age at first birth after adjusting for parity [58]. The finding of a reduced risk of death from asthma associated with higher parity is not in agreement with a previous study conducted in Australia, which reported an increased risk of later-onset asthma associated with higher parity [59]. Jenkins et al. reported that the risk of later-onset asthma among women who had no asthma by age seven was associated with increased parity. However, the risk of asthma decreased with increasing age at first birth [59].

Psychological and social factors were also identified as important contributing factors in asthma deaths [60, 61]. Bucknall et al., analyzing 95 deaths attributable to asthma, found psychosocial problems in 58 % of cases; the commonest problems were depression and denial of symptoms or of the need of treatment [61].

In a small number of cases, thunderstorms in the pollen season have been identified as triggers of severe asthma exacerbations. The main hypotheses explaining association between thunderstorms and asthma claim that thunderstorms may concentrate pollen grains at ground level which may then release allergenic particles of respirable size in the atmosphere after their rupture by osmotic shock, which in turn may induce asthmatic reactions, often severe [62–65].

### Avoidable factors in asthma deaths

Over the past 50 years several studies have repeatedly identified potentially preventable factors in the majority of asthma deaths. Most of these factors deal with the management of asthma. Inadequate education of patients on recognising risk and the appropriate action needed when asthma control is poor, deficiencies in the accuracy and timing of asthma diagnosis, inadequate classification of severity and treatment, seem to play a part in the majority of asthma deaths [20, 21].

Recently a confidential enquiry in the United Kingdom, the “National Review of Asthma Deaths” (NRAD), has reported that deaths from asthma were avoidable in over 60 % of 195 cases of asthma deaths studied [20]. The NRAD identified 195 cases of asthma deaths in the United Kingdom during 2012–2013 [20]. Key findings from the report include that nearly half died without seeking medical assistance or before emergency medical care could be provided, and the majority were not under specialist medical supervision during the year prior to death [18, 19]. Only one-quarter had been provided with a personal asthma action plan, acknowledged to improve asthma care, and there was evidence of excessive prescribing of short-acting reliever medication, under-prescribing of preventer medication, and inappropriate prescribing of long-acting beta-agonist bronchodilator inhalers as the sole form of treatment. Overall asthma management (acute and chronic) was satisfactory in only 31 % out of 195 people who died [20].

## Conclusions

Death from asthma is a complex phenomenon and represents the “tip of the iceberg” with respect to the global burden of asthma. Although asthma mortality rates have declined in many high-income countries, continued surveillance of asthma mortality rates is essential to monitor progress in asthma care. Despite major advances in the treatment, asthma still kills.

Preventable factors have been identified in the majority of asthma deaths. Inadequate education of patients on recognising risk and the appropriate action needed when asthma control is poor, deficiencies in the accuracy and timing of asthma diagnosis, inadequate classification of severity and treatment, seem to play a part in the majority of asthma deaths.

Improvements in management, epitomized by the use of guided self-management systems of care may be the key goals in reducing asthma mortality worldwide.

## Abbreviations

NFA: Near fatal asthma
ICS: Inhaled corticosteroids

## References

[1] Global Initiative for Asthma (GINA). The Global Strategy for Asthma Management and Prevention. GINA, 2014. www.ginasthma.org.

[2] The Global Asthma report 2014, www.globalasthmareport.org.

[3] World Health Organization (2007). Global surveillance, prevention and control of chronic respiratory diseases: a comprehensive approach.

[4] Chung KF, Wenzel SE, Brozek JL, Bush A, Castro M, Sterk PJ. International ERS/ATS guidelines on definition, evaluation and treatment of severe asthma. Eur Respir J. 2014;43(2):343–73. doi:10.1183/09031936.00202013. Epub 2013 Dec 12. Erratum in: Eur Respir J. 2014;43(4):1216. 10.1183/09031936.00202013 24337046

[5] EUROSTAT. European shortlist: cause of death statistics. http://epp.eurostat.ec.europa.eu/statistics_explained/index.php/Glossary:European_shortlist_of_causes_of_death Date last updated: April, 2014 Date last accessed: June 2, 2014.

[6] Documento di Strategia – GARD Italia 2009, www.salute.gov.it.

[7] Ulrik CS, Frederiksen J (1995). Mortality and markers of risk of asthma death among 1,075 outpatients with asthma. Chest.

[8] American Lung Association Epidemiology and Statistics Unit Research and Health Education Division (2012). Trends in Asthma Morbidity and Mortality.

[9] Centers for Disease Control and Prevention, National Center for Health Statistics (2012). CDC Wonder On-line Database, compiled from Compressed Mortality File 1999-2009 Series 20 No. 2O.

[10] Centers for Disease Control and Prevention. National Center for Health Statistics. National Hospital Discharge Survey, 1989-2010. Analysis performed by American Lung Association Research and Health Education Division using SPSS software.

[11] Statistics and Information Department, Minister’s Secretariat, Ministry of Health and Welfare, Japan. Vital Statistics Japan 3. Tokyo: Health and Welfare Statistics Association; 2012–2013.

[12] Ito Y, Tamakoshi A, Wakai K, Takagi K, Yamaki K, Ohno Y. Trends in asthma mortality in Japan. J Asthma. 2002;39(7):633–9. 10.1081/jas-120014928 12442953

[13] Kaur BP, Lahewala S, Arora S, Agnihotri K, Panaich SS, Secord E, et al. Asthma: hospitalization trends and predictors of in-hospital mortality and hospitalization costs in the USA (2001–2010). Int Arch Allergy Immunol. 2015;168(2):71–8. doi:10.1159/000441687. Epub 2015 Nov 24. 10.1159/000441687 26595589

[14] Wijesinghe M, Weatherall M, Perrin K, Crane J, Beasley R. International trends in asthma mortality rates in the 5- to 34-year age group: a call for closer surveillance. Chest. 2009;135(4):1045–9. doi:10.1378/chest.08-2082. 10.1378/chest.08-2082 19349400

[15] Ali Z, Dirks CG, Ulrik CS (2013). Long-term mortality among adults with asthma: a 25-year follow-up of 1,075 outpatients with asthma. Chest.

[16] Bellia V, Pedone C, Catalano F, Zito A, Davì E, Palange S, et al. Asthma in the elderly: mortality rate and associated risk factors for mortality. Chest. 2007;132(4):1175–82. Epub 2007 Sep 21. 10.1378/chest.06-2824 17890479

[17] Sheikh A, Steiner MF, Cezard G, Bansal N, Fischbacher C, Simpson CR, et al, Ethnic variations in asthma hospital admission, readmission and death: a retrospective, national cohort study of 4.62 million people in Scotland. BMC Med. 2016;14:3. doi:10.1186/s12916-015-0546-6. 10.1186/s12916-015-0546-6 PMC4710027 26755184

[18] Boulet LP (2014). Is asthma control really more difficult to achieve in the elderly patient?. Int Arch Allergy Immunol.

[19] Fleming DM, Cross KW, Sunderland R, Ross AM (2000). Comparison of the seasonal patterns of asthma identified in general practitioner episodes, hospital admissions, and deaths. Thorax.

[20] Royal College of Physicians (2014). Why Asthma Still Kills:the National Review of Asthma Deaths (NRAD) Confidential Enquiry Report.

[21] Buist AS (1988). Is asthma mortality increasing [editorial]?. Chest.

[22] Levy ML (2015). The national review of asthma deaths: what did we learn and what needs to change?. Breathe.

[23] Selroos O, Kupczyk M, Kuna P, Łacwik P, Bousquet J, Brennan D, et al. National and regional asthma programmes in Europe. Eur Respir Rev. 2015;24(137):474–83. doi:10.1183/16000617.00008114. 10.1183/16000617.00008114 PMC9487682 26324809

[24] Royal College of Physicians and Royal College of Pathologists (1982). Medical aspects of death certification. J R Coll Physicians Lond.

[25] Suisa S, Ernst P. Inhaled corticosteroids: impact on asthma morbidity and mortality. J Allergy Clin Immunol. 2001;107:937–44. doi:10.1067/mai.2001.115653. 10.1067/mai.2001.115653 11398069

[26] Bisgaard H, Hermansen MN, Loland L, Halkjaer LB, Buchvald F. Intermittent inhaled corticosteroids in infants with episodic wheezing. N Engl J Med. 2006;11(354):1998–2005. doi:10.1056/NEJMoa054692. 10.1056/NEJMoa054692 16687712

[27] Nelson HS. Is there a problem with inhaled long-acting beta-adrenergic agonists? J Allergy Clin Immunol. 2006;2(1):3–16. doi:10.1016/j.jaci.2005.10.013. 10.1016/j.jaci.2005.10.013 16387577

[28] Sears MR (2002). Adverse effects of beta-agonists. J Allergy Clin Immunol.

[29] Weatherall M, Wijesinghe M, Perrin K, Harwood M, Beasley R. Meta-analysis of the risk of mortality with salmeterol and the effect of concomitant inhaled corticosteroid therapy. Thorax. 2010;65(1):39–43. doi:10.1136/thx.2009.116608. 10.1136/thx.2009.116608 20029037

[30] Suissa S, Ernst P, Boivin JF, Horwitz RI, Habbick B, Cockroft D, et al. A cohort analysis of excess mortality in asthma and the use of inhaled beta-agonists. Am J Respir Crit Care Med. 1994;2(3):604–10. doi:10.1164/ajrccm.149.3.8118625. 10.1164/ajrccm.149.3.8118625 8118625

[31] Sturdy PM, Victor CR, Anderson HR, Bland JM, Butland BK, Harrison BD, et al. Psychological, social and health behaviour risk factors for deaths certified as asthma: a national case-control study. Thorax. 2002;57:1034–9. 10.1136/thorax.57.12.1034 PMC1758792 12454297

[32] Turner MO, Noertjojo K, Vedal S, Bai T, Crump S, FitzGerald JM (1998). Risk factors for near-fatal asthma. A case-control study in hospitalized patients with asthma. Am J Respir Crit Care Med.

[33] Pumphrey RSH, Gowland MH (2007). Further fatal allergic reactions to food in the United Kingdom, 1999-2006. J Allergy Clin Immunol.

[34] Alvarez GG, Schulzer M, Jung D, Fitzgerald JM (2005). A systematic review of risk factors associated with near-fatal and fatal asthma. Can Respir J.

[35] Suissa S, Blais L, Ernst P (1994). Patterns of increasing beta-agonist use and the risk of fatal or near- fatal asthma. Eur Respir J.

[36] Roberts G, Patel N, Levi-Schaffer F, Habibi P, Lack G (2003). Food allergy as a risk factor for life-threatening asthma in childhood: a case-controlled study. J Allergy Clin Immunol.

[37] D’Amato G, Vitale C, Lanza M, Sanduzzi A, Molino A, Mormile M, et al. Near fatal asthma: treatment and prevention. Eur Ann Allergy Clin Immunol. 2016;48(N4):116–22. 27425166

[38] Restrepo RD, Peters J (2008). Near-fatal asthma: recognition and management. Curr Opin Pulm Med.

[39] Richards GN, Kolbe J, Fenwick J, Rea HH (1993). Demographic characteristics of patients with severe life threatening asthma: comparison with asthma deaths. Thorax.

[40] Zureik M, Neukirch C, Leynaert B, Liard R, Bousquet J, Neukirch F (2002). Sensitisation to airborne moulds and severity of asthma: cross sectional study from European Community respiratory health survey. BMJ.

[41] Denning DW, O’Driscoll BR, Hogaboam CM, Bowyer P, Niven RM (2006). The link between fungi and severe asthma: a summary of the evidence. Eur Respir J.

[42] O’Hollaren MT, Yunginger JW, Offord KP, Somers MJ, O’Connell EJ, Ballard DJ, et al. Exposure to an aeroallergen as a possible precipitating factor in respiratory arrest in young patients with asthma. N Engl J Med. 1991;324(6):359–63. 10.1056/NEJM199102073240602 1987459

[43] Black PN, Udy AA, Brodie SM (2000). Sensitivity to fungal allergens is a risk factor for life-threatening asthma. Allergy.

[44] Targonski PV, Persky VW, Ramekrishnan V (1995). Effect of environmental molds on risk of death from asthma during the pollen season. J Allergy Clin Immunol.

[45] O’Driscoll RB, Hopkinson L, Denning DW (2005). Mold sensitisation allergy is common amongst patients with severe asthma requiring multiple hospital admissions. BMC Pulm Med.

[46] Halonen M, Stern DA, Wright AL, Taussig LM, Martinez FD. Alternaria as a major allergen for asthma in children raised in a desert environment. Am J Respir Crit Care Med. 1997;155:1356–61. 10.1164/ajrccm.155.4.9105079 9105079

[47] Nelson HS, Szefler SJ, Jacobs J, Huss K, Shapiro G, Sternberg AL. The relationships among environmental allergen sensitization, allergen exposure, pulmonary function and bronchial hyperresponsiveness in the Childhood Asthma Management Program. J Allergy Clin Immunol. 1999;104:775–85. 10.1016/s0091-6749(99)70287-3 10518821

[48] Asher MI, Keil U, Anderson HR, Beasley R, Crane J, Martinez F, et al. International Study of Asthma and Allergies in Childhood (ISAAC): rationale and methods. Eur Respir J. 1995;8(3):483–91. 10.1183/09031936.95.08030483 7789502

[49] Greiling AK, Boss LP, Wheeler LS (2005). A preliminary investigation of asthma mortality in schools. J Sch Health.

[50] Becker JM, Rogers J, Rossini G, Mirchandani H, D’Alonzo GE (2004). Asthma deaths during sports: report of a 7-year experience. J Allergy Clin Immunol.

[51] Almind M, Viskum K, Evald T, Dirksen A, Kok-Jensen A. A seven-year follow-up study of 343 adults with bronchial asthma. Dan Med Bull. 1992;39(6):561–5. 1468265

[52] Huovinen E, Kaprio J, Vesterinen E, Koskenvuo M (1997). Mortality of adults with asthma: a prospective cohort study. Thorax.

[53] Zoratti EM, Havstad S, Rodriguez J, Robens-Paradise Y, Lafata JE, McCarthy B (1998). Health service use by African Americans and Caucasians with asthma in a managed care setting. Am J Respir Crit Care Med.

[54] Clement LT, Jones CA, Cole J (2008). Health disparities in the United States:childhood asthma. Am J Med Sci.

[55] Hill TD, Graham LM, Divgi V (2011). Racial disparities in pediatric asthma: a review of the literature. Curr Allergy Asthma Rep.

[56] Martinez FD, Vercelli D. Asthma. Lancet. 2013;382:1360–72. 10. Patient-Centered Outcomes Research Institute. http://www.pcori.org/content/ pcori-approves-23-million-research-reduce-disparities-asthma-burden-andoutcomes. Accessed 28 June 2015.

[57] Netuveli G, Hurwitz B, Levy M, Fletcher M, Barnes G, Durham SR (2005). Ethnic variations in UK asthma frequency, morbidity, and health-service use: a systematic review and meta-analysis. Lancet.

[58] Chen CC, Chiu HF, Yang CY (2014). Parity, age at first birth, and risk of death from asthma: evidence from a cohort in taiwan. Int J Environ Res Public Health.

[59] Jenkins MA, Dharmage SC, Flander LB, Douglass JA, Ugoni AM, Carlin JB, et al. Parity and decreased use of oral contraceptives as predictors of asthma in young women. Clin Exp Allergy. 2006;36:609–13. 10.1111/j.1365-2222.2006.02475.x 16650045

[60] Bucknall CE, Slack R, Godley CC, Mackay TW, Wright SC (1999). Scottish Confidential Inquiry into Asthma Deaths (SCIAD), 1994–6. Thorax.

[61] Yellowlees PM, Kalucy RS (1990). Psychobiological aspects of asthma and the consequent research implications. Chest.

[62] D’Amato G, Vitale C, D’Amato M, Cecchi L, Liccardi G, Molino A, et al. Thunderstorm-related asthma: what happens and why. Clin Exp Allergy. 2016;46(3):390–6. doi:10.1111/cea.12709. 10.1111/cea.12709 26765082

[63] D’Amato G, Corrado A, Cecchi L, Liccardi G, Stanziola A, Annesi-Maesano I, et al. A relapse of near-fatal thunderstorm-asthma in pregnancy. Eur Ann Allergy Clin Immunol. 2013;45(3):116–7. 23862404

[64] D’Amato G, Pawankar R, Vitale C, Lanza M, Molino A, Stanziola A, et al. Climate change and air pollution: effects on asthma and respiratory allergy. Allergy Asthma Immunol Res. 2016;8(5):391–5. 10.4168/aair.2016.8.5.391 PMC4921692 27334776

[65] D’Amato G, Holgate ST, Pawankar R, Ledford DK, Cecchi L, Al-Ahmad M, et al. Meteorological conditions, climate change, new emerging factors, and asthma and related allergic disorders. A statement of the World Allergy Organization. World Allergy Organ J. 2015;8(1):25. doi:10.1186/s40413-015-0073-0. 10.1186/s40413-015-0073-0 PMC4499913 26207160

